# Respiratory syncytial virus B sequence analysis reveals a novel early genotype

**DOI:** 10.1038/s41598-021-83079-2

**Published:** 2021-02-10

**Authors:** Juan C. Muñoz-Escalante, Andreu Comas-García, Sofía Bernal-Silva, Daniel E. Noyola

**Affiliations:** 1 Microbiology Department, Facultad de Medicina, Universidad Autónoma de San Luis Potosí, Avenida Venustiano Carranza 2405, San Luis Potosí, 78210 México; 2 Center for Research in Biomedicine and Health Sciences, Facultad de Medicina, Universidad Autónoma de San Luis Potosí, San Luis Potosí, Mexico

**Keywords:** Virology, Viral epidemiology, Viral infection

## Abstract

Respiratory syncytial virus (RSV) is a major cause of respiratory infections and is classified in two main groups, RSV-A and RSV-B, with multiple genotypes within each of them. For RSV-B, more than 30 genotypes have been described, without consensus on their definition. The lack of genotype assignation criteria has a direct impact on viral evolution understanding, development of viral detection methods as well as vaccines design. Here we analyzed the totality of complete RSV-B G gene ectodomain sequences published in GenBank until September 2018 (n = 2190) including 478 complete genome sequences using maximum likelihood and Bayesian phylogenetic analyses, as well as intergenotypic and intragenotypic distance matrices, in order to generate a systematic genotype assignation. Individual RSV-B genes were also assessed using maximum likelihood phylogenetic analyses and multiple sequence alignments were used to identify molecular markers associated to specific genotypes. Analyses of the complete G gene ectodomain region, sequences clustering patterns, and the presence of molecular markers of each individual gene indicate that the 37 previously described genotypes can be classified into fifteen distinct genotypes: BA, BA-C, BA-CC, CB1-THB, GB1-GB4, GB6, JAB1-NZB2, SAB1, SAB2, SAB4, URU2 and a novel early circulating genotype characterized in the present study and designated GB0.

## Introduction

Respiratory syncytial virus (RSV) is a leading cause of lower respiratory tract infections in infants, elderly adults, and immunosuppressed individuals^1^. Since the discovery of RSV, a wide diversity of viral strains has been identified leading to the classification in two major groups (RSV-A and RSV-B), as well as multiple genotypes^2–4^. RSV infections occur worldwide and co-circulation of viral strains from both major groups is common^5^. RSV-B strains are the predominant viruses in approximately one third of winter seasons^6,7^. Since the initial description of RSV genotypes, there has been an increasing number of reported genotypes, with worldwide extension of novel strains and apparent extinction of older types. The diversity in genotypes of RSV-B strains is greater than for RSV A. While RSV-A strains can be grouped into seven distinct genotypes, there have been at least 37 RSV-B genotypes described in the literature (GB1, GB2, GB3, GB4, GB5, GB6, GB12, GB13, SAB1, SAB2, SAB3, SAB4, URU1, URU2, CB1, THB, BA1, BA2, BA3, BA4, BA5, BA6, BA7, BA8, BA9, BA10, BA11, BA12, BA13, BA14, BA-Ly, BA-C, BA-CCA, BA-CCB, JAB1, NZB1, and NZB2)^4,9–27^. Of note, at present there is no consensus regarding criteria to discriminate between genotypes^4,8,17,20,28,29^. Identification and description of many genotypes has relied on sequencing of the second hypervariable region of the G gene; however, analyses limited to this region are not always able to distinguish between strains that might be considered as part of the same or different genotype^8,30^. As a result, some viral clusters which have been described as distinct genotypes have turned out to belong to previously identified genotypes. Classification of viral strains is of relevance not only for taxonomic purposes, but in order to better understand the epidemiology of this important virus, as well as the development of therapeutic and preventive strategies. In the present work we have analyzed a large set of RSV-B sequences using a methodology previously described for RSV-A genotype analysis^8^. Our results indicate that many of the 37 previously described genotypes can be reorganized within a smaller number of genotypes based on intra and inter-clade variability. In addition, a previously unrecognized RSV-B cluster composed of strains that circulated between 1972 and 1983 shows unique characteristics enough to identify them as a distinct early genotype.

## Results

### Dataset selection

From a total of 10,340 RSV sequences downloaded from NCBI, 3029 corresponded to the RSV-B complete G gene ectodomain; nevertheless, 831 sequences (27.4%) were discarded due to indels or degenerate nucleotides that could interfere in a correct genotype assignation (Supplementary Fig. 1). The final dataset comprised 2198 sequences with at least the complete G gene ectodomain and was used for genotypes analysis. This dataset included 1673 sequences of only complete G gene ectodomain, 18 sequences (0.82%) of only the complete G gene, 29 sequences (1.32%) of only SH and G genes, and 478 complete genome sequences. For all the sequences, the complete G gene ectodomain was used to assign genotypes; sequences with one or more complete genes were used for cladistic analyses and detection of molecular markers at nucleotide and amino acid sequences.

### Genotype assignment

**Figure 1:**
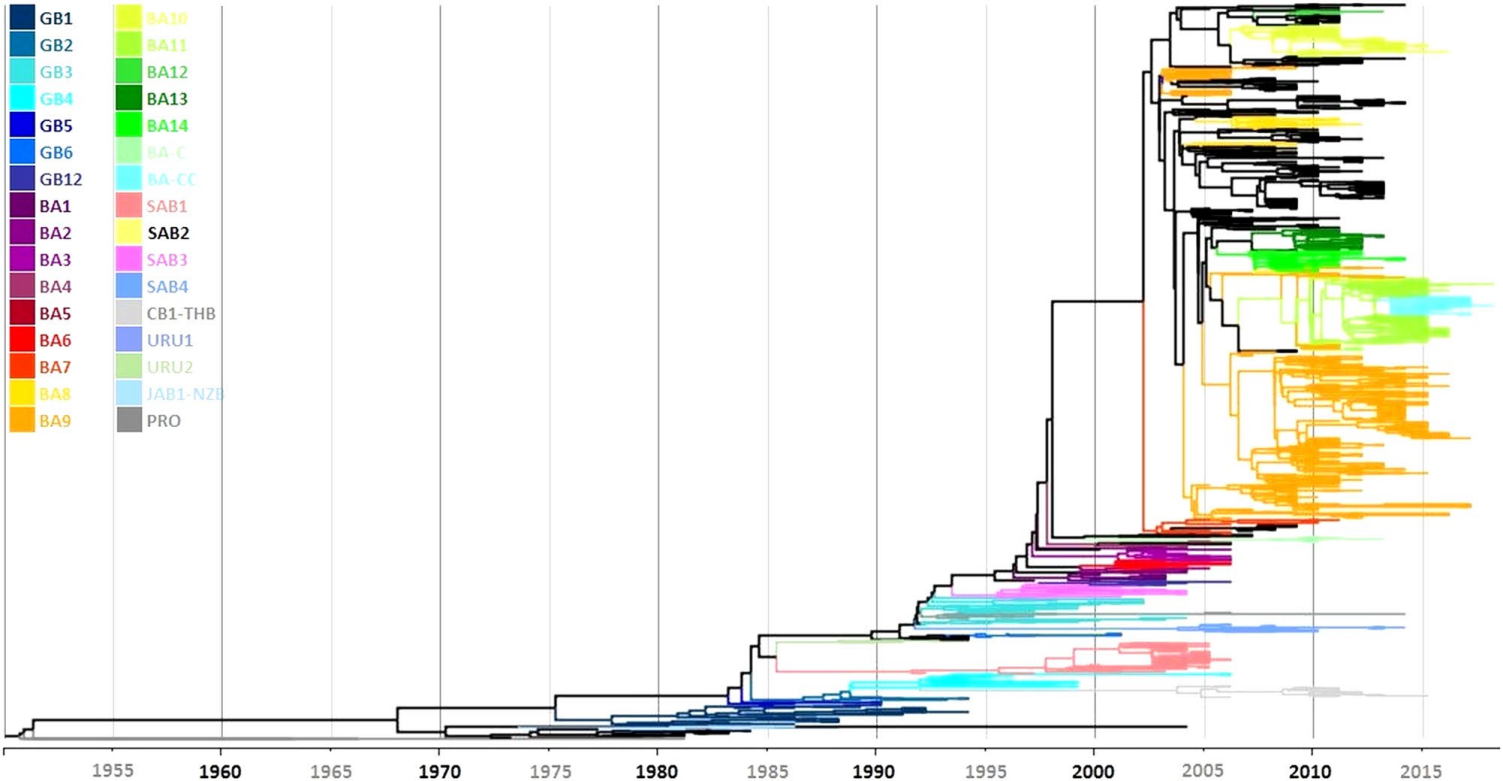
Phylogenetic tree of 1,334 unique RSV-B complete G gene ectodomain sequences constructed by Bayesian MCMC analysis. Genotype assignment was carried out with the use of 169 reference sequences including 37 previously described genotypes and prototype strains.

Genotype assignment was carried out by clustering of 1,334 unique RSV-B complete G gene ectodomain sequences (corresponding to 60.7% of the dataset) with sequences previously designated as reference sequences or with equivalent reference sequences, defined as described in the Methods section, using both Maximum Likelihood method and Bayesian MCMC. Clade distribution, topology and clustering of sequences were concordant in both methods (Fig. 1 and Supplementary Fig. 2).

The largest number of sequences corresponded to the BA9 genotype (27.61%), followed by SAB1 (7.11%), and BA11 (5.05%); all other genotypes contributed with less than 2.5% each. Of note, CB1 and THB genotypes previously described by Cui et al.^15^ and Auksornkitti et al.^25^, JAB1 and NZB2 previously described by Kuroiwa et al.^18^ and Matheson et al.^19^, and BA-CCA and BA-CCB genotypes described by Gaymard et al.^13^ clustered and intermingled in individual clades, suggesting that each couple of genotypes correspond to the same genotype (hereafter referred to as CB1-THB, JAB1-NZB2, and BA-CC), which was corroborated during molecular marker analysis as described below. Ten well defined and sustained clades including two or more sequences did not cluster with any reference or equivalent reference sequences and were assigned as unidentified clades (U1-10); all these clades, except U1, had the 60-nucleotide duplication characteristic of BA strains. Eight sequences did not cluster with any other sequence either within previously described genotypes or unidentified clades; because of this, they were considered as singletons and were excluded from subsequent genotype analyses.

To corroborate the genotype assignment, an intergenotypic and intragenotypic p-distance matrix was generated with all the sequences (n = 2190) which were assigned to previously described genotypes (n = 37) and unidentified clades (n = 10). GB1 presented the highest intragenotypic distance (p = 0.0358) and this value was used as the threshold to identify clades which belong to the same or different genotype (Supplementary Fig. 3).

**Figure 2:**
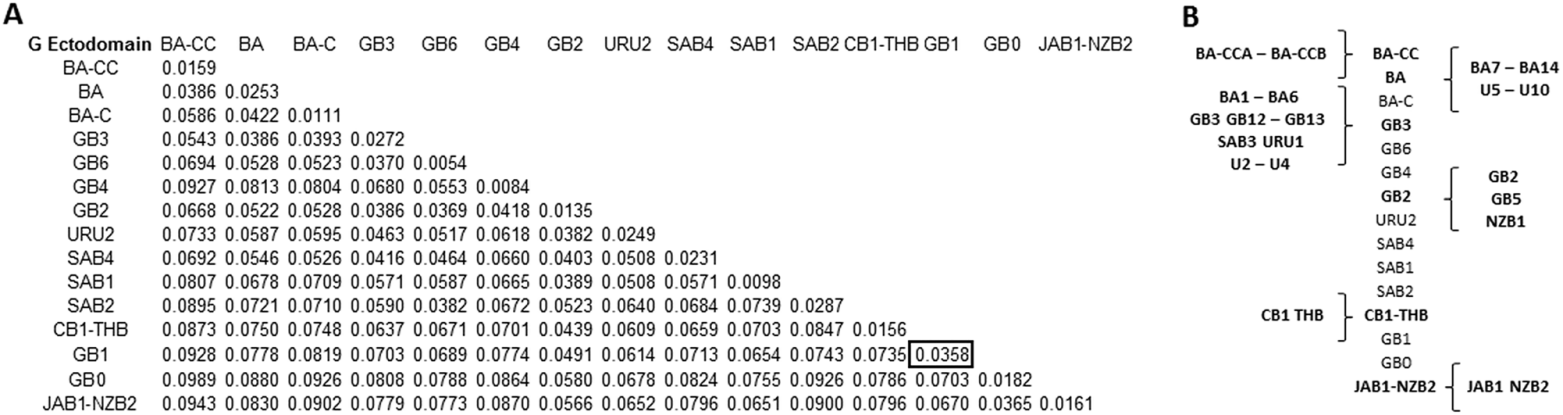
(**A**) Distinct genotypes identified through intergenotypic and intragenotypic p-distance analysis of 2,190 RSVB complete ectodomain sequences. The highest intragenotypic distance was observed for GB1 (0.0358). All clusters with intergenotypic distance higher than this threshold value were considered as distinct genotypes. (**B**) Several previously described genotypes or unique unidentified clusters were found to cluster together with BA-CC (BA-CCA and BA-CCB), CB1-THB (CB1 and THB), JAB1-NZB2 (JAB1 and NZB2), GB2 (GB2, GB5, and NZB1), GB3 (GB3, GB12-GB13, SAB3, URU1, BA1-BA6, U2-U4), and BA (BA7-BA14, U5-U10).

**Figure 3:**
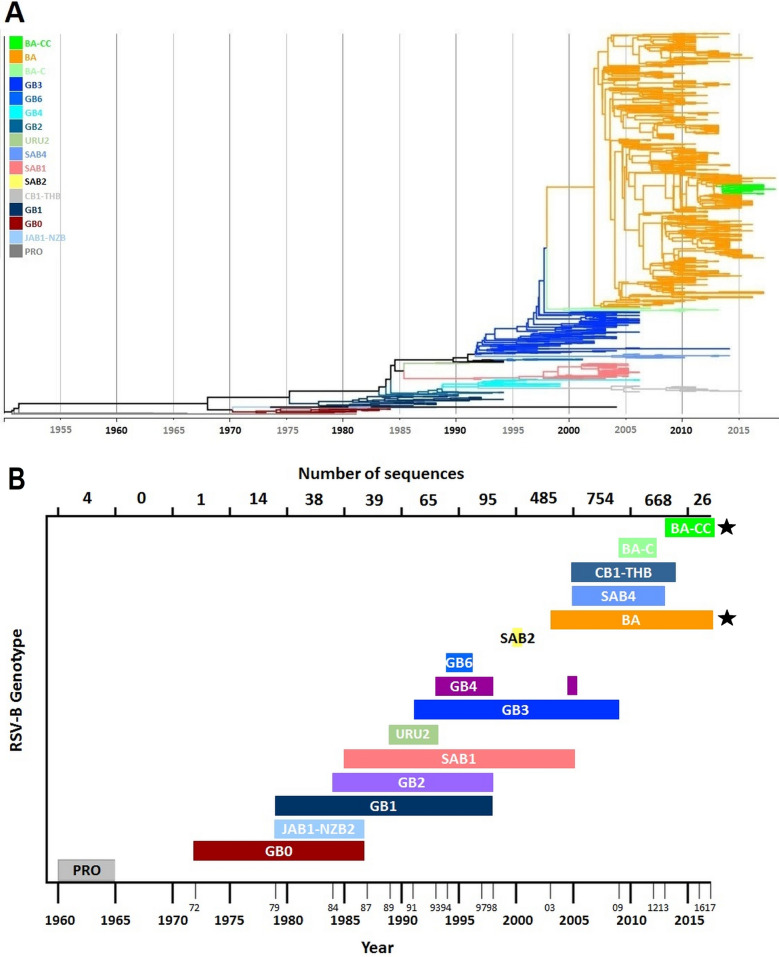
(**A**) Phylogenetic tree of 1,334 unique RSV-B complete G gene ectodomain sequences constructed by Bayesian MCMC analysis showing the 15 distinct genotypes defined through intragenotypic and intergenotypic p-distance analysis. (**B**) Temporal distribution of RSV-B genotypes since their first up to their last detection. Genotypes marked with stars indicate genotypes currently in circulation.

Genotypes and unidentified clades were grouped following a stepwise lowest distance neighbor joining strategy until all groups distance were higher than the threshold. This resulted in the joining of genotypes GB2, GB5 and NZB1 in a single genotype designated as genotype GB2; the joining of genotypes GB3, GB12, GB13, SAB3, URU1, BA1-6 and clades U2-4 into a single genotype designated as genotype GB3; and the joining of genotypes BA7-14 and clades U5-U10, designated as genotype BA (Fig. 2). Remarkably, an independent clade (U1) with sequences from strains isolated up to seven years (1972) before isolation of the first GB1 sequence included in the dataset (1979) was identified; this clade was subsequently assigned as genotype GB0 (Fig. 3; Supplementary Fig. 4). The lowest intergenotypic p-distance between this clade and the rest of genotypes was 0.0578 (when compared with GB2), a value exceeding by 1.6 times the threshold value of 0.0358 (Fig. 2). Sequences included in the GB0 genotype clade have not been described to conform a unique genotype previously.

### Complete NS1, NS2, N, P, M, SH, G, F, M2, and L gene analysis

**Figure 4:**
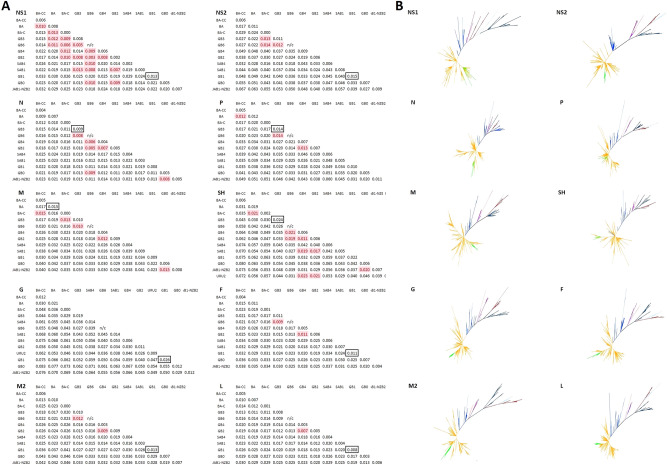
(**A**) Intergenotypic and intragenotypic p-distance analysis of complete NS1, NS2, N, P, M, SH, G, F, M2, and L genes of RSV-B sequences. (**B**) Unrooted phylogenetic tree of complete NS1, NS2, N, P, M, SH, G, F, M2, and L genes of RSV-B sequences constructed Maximum Likelihood analysis.

For each of the ten RSV-B genes (NS1, NS2, N, P, M, SH, G, F, M2, and L) cladograms were generated from the corresponding Maximum Likelihood analysis under the best fitting substitution model for each gene dataset, as well as the corresponding intergenotypic and intragenotypic p-distance matrices based on the previously assigned genotypes (Fig. 4). Genotypes SAB2 and CB1-THB were not included on individual gene analysis due to lack of complete genes or complete genomes sequences for these genotypes; URU2 was only included on complete SH and G genes analysis due the presence of only partial genome sequences for this genotype.

Cladogram topologies and sequence clustering was concordant in the majority of genes; sequences assigned to a specific genotype grouped in well differentiated clusters, with exception of the recently identified genotype designated as BA-CC. Sequences assigned as BA-CC grouped on two different but proximate clades in the NS1 gene cladogram. Furthermore, p-distance value analysis showed concordant genotype assignation for most genotypes in most gene matrices, with NS1 matrix being the exception with 19 discordances. Overall, 50 (7.3%) of the 684 intergenotypic comparisons had p values lower than the threshold. This was partly explained by the small number of sequences available for some genotypes; for instance, for comparisons for which there were 20 sequences or less the proportion of intergenotypic p-values lower than the threshold was higher (30 of 275 instances, 10.9%) than for comparisons for which there were more than 20 sequences available (20 of 409 instances, 4.9%; P = 0.003). Of note, GB6 (for which there was only one full genome sequence) was included as one of the genotypes in 20 (40%) of the 50 comparisons in which the p-value was below the threshold.

All RSV genes datasets were assessed for recombination with RDP, GENECONV, Chimaera, MAxChi, BootScan, SiScan and 3Seq algorithms using RDP4 v.4.100^31^, as well as GARD algorithm^32^. There was no evidence of recombination among RSV sequences included in the study.

### Molecular markers detection

For each of the ten RSV-B genes (NS1, NS2, N, P, M, SH, G, F, M2, and L), nucleotide sequences spanning from 3′UTR to 5′UTR were aligned and grouped in accordance with genotype assignment. Each genotype was compared against RSV-B reference sequence “strain B1” (Accession Number NC_001781.1) and every variant at every site was recorded. Amino acid sequences were deduced from each of the ten RSV-B coding regions, and variants were recorded as previously described^8^. Variants fixed in more than 75% of the genotype sequences were considered as molecular markers.

**Figure 5:**
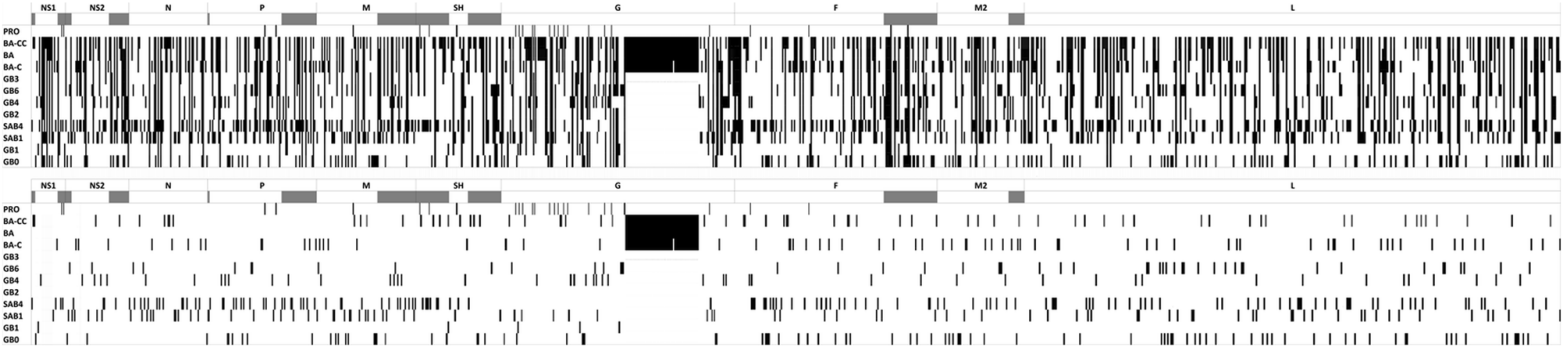
Distribution of molecular markers present in RSV-B genotypes. The location of all (upper panel) and unique (lower panel) molecular markers present in each genotype is shown. Dark bars indicate untranslated regions in which molecular markers were identified.

In total, 1,213 nucleotide variants distributed at the total length of the genome fulfilled the criteria to be considered molecular markers; 636 (52.4%) of them were present in a single genotype (Fig. 5 and Table 1). In addition, 213 deduced amino acid variants at the total of proteins fulfilled the criteria of molecular markers; 107 (50.2%) of these molecular markers were present in a single genotype. For genotypes GB2, GB3, and BA no unique molecular markers were detected. Genotype GB0 had 71 nucleotide molecular markers and 8 amino acid molecular markers which were unique for this genotype (Fig. 5, Table 1, and Supplementary Table 1).

**Table 1:** Number of unique and shared nucleotide and amino acid molecular markers identified in each RSV-B genotype.

Genotype	Nucleotide molecular markers			Amino acid molecular markers		
	Unique	Shared	Total	Unique	Shared	Total
BA-CC	71	369	440	10	81	91
BA	0	374	374	0	81	81
BA-C	73	338	411	11	73	84
GB3	0	273	273	0	48	48
GB6	44	280	324	7	53	60
GB4	46	246	292	15	42	57
GB2	0	232	232	0	38	38
SAB4	142	277	419	18	46	64
SAB1	97	226	323	21	47	68
GB1	3	84	87	2	22	24
GB0	71	198	268	8	36	44
JAB1-NZB2	89	176	265	15	32	47
All genotypes	636	577	1,213	107	106	213

### Geographic and temporal distribution

**Figure 6:**
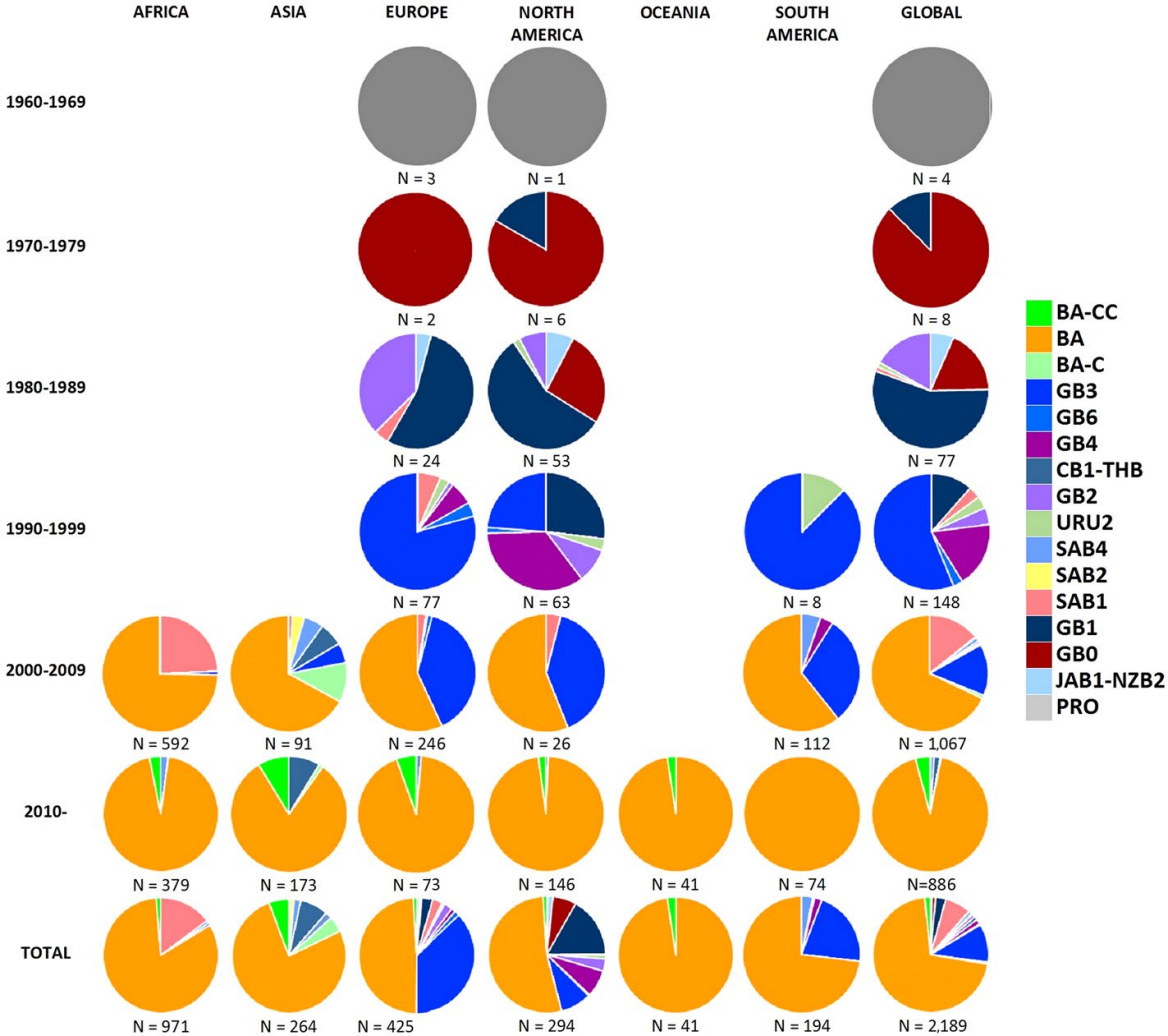
RSV-B genotype distribution since 1960 and each decade thereafter, according to continent of viral detection.

Date and country (continent) of isolation of the strain corresponding to each sequence in the dataset was recorded. As noted previously, eight (0.36%) of the 2198 sequences in the dataset were not assigned to any genotype. Geo-temporal records showed circulation of GB0 starting in 1972, seven years later than the last Prototype RSV-B sequence was isolated in Europe. This was the only genotype detected up to 1979, when GB1 was first isolated; these two genotypes co-circulated until the mid-80s. Around this time, an initial diversification event occurred, with the emergence of JAB1-NZB2, SAB1, URU2, and GB2 genotypes (Figs. 3B and 6). A second diversification event occurred in the mid-90s, leading to the appearance of GB3, GB4, and GB6 genotypes, and characterized by a global spread and predominance of GB3. Of interest, the dataset included five GB3 sequences isolated in the United States obtained from RSV strains isolated between 1996 and 1998 which display the 60-nucleotide duplication described in 2003^33^. These sequences did not group in a single cluster, but were present in four different clades. Furthermore, the sequence of the duplicated region of these early strains showed nucleotide and amino acid differences compared with the initial BA strains described in Buenos Aires, Argentina; nucleotide differences between these GB3 and the original BA viruses were also noted in other genes (NS1, N, P, M, SH, F, M2, and L). After the year 2000, the emergence of CB1-THB occurred; this genotype derived from GB2 and does not have the 60-nucleotide duplication. In addition, during this time the BA-C genotype derived from GB3, containing the partial duplication in the G-gene. These two genotypes have shown an apparent geographic circulation limited to the Asian continent. In contrast, the BA genotype, derived from GB3 sequences with the 60-nucleotide duplication, spread globally and became the predominant genotype worldwide. Finally, the most recent genotype (BA-CC) emerged after 2010 showing a global distribution.

## Discussion

Over the last decades, a large number of RSV-B genotypes have been reported in the literature^9–15,23,25,26^. Genotype description is usually based on analysis of a fragment of the G gene of this virus. However, there is no consensus regarding criteria that should be met in order to identify a new genotype, particularly the size of the G gene sequence that needs to be analyzed and the number of sequences that should be included in the analysis^8,20,28,29^. Of relevance, many studies have included a limited number of sequences and have analyzed only the hypervariable region of the G gene. As a result, RSV strains that have been described as distinct genotypes might group in the same cluster when analyzed together^15,25^. These observations, together with the increasing number of sequence data available, underscore the advantage of a systematic approach for viral classification.

Using the approach that we recently described for RSV-A genotype analysis, we analyzed 2,198 sequences of the RSV-B G gene sequence encoding for the complete G protein ectodomain and found that when all available sequences (as of September, 2018) were analyzed, many of previously described genotypes cluster with other strains previously considered as unique genotypes. As such, the number of distinct RSV-B genotypes identified through this analysis was fifteen (in contrast to at least 37 genotypes mentioned in the literature). Analysis of full sequences of each of the complete genes from 478 RSV-B strains for which the complete genome sequence was available supported the inclusion of many previously described genotypes within a smaller number of genotypes. It is noteworthy that although analysis of other genes did not allow for distinction between all genotypes in all comparisons, in many instances this result can be explained by the paucity of sequences available for some RSV-B genotypes. For instance, there was only one full genome sequence available for the GB6 genotype, with comparisons that include this genotype accounting for 20 (40%) of the 50 instances in which intergenotypic p-distance did not support classification of a specific clade as a distinct genotype. Overall, 30 (10.9%) of the 275 intergenotypic comparisons that included 20 sequences or less did not allow to differentiate between genotypes in contrast to 4.9% of comparisons that included more than 20 sequences. This highlights that inclusion of a minimum number of sequences of each genotype might be required for definite genotype assignment.

One of the most notable distinct features of some RSV-B genotypes is the presence of a partial duplication of the G gene, initially described in Buenos Aires, Argentina, and termed BA strains^33^. Over the past two decades, RSV-B strains harboring this partial duplication have become the predominant RSV-B viruses. As a result of global expansion and diversification, a large number of BA genotypes have been described. Intra- and intergenotypic comparisons between BA genotypes indicated that these strains can be classified within four distinct groups: early BA strains (which clustered within the GB3 genotype), late BA strains, BA-C, and BA-CC genotypes. While the partial duplication of the G gene is the landmark characteristic of BA strains, the p-distance between early BA strains and GB3 strains was below the threshold to consider them distinct genotypes. This result is similar to analysis of RSV-A strains with a partial duplication of the G gene (ON1 strains) which also present a low p-distance compared to RSV-A viruses without the duplication (NA1 genotype) and, as a result, have been considered as part of the NA1 genotype^8,30^. In addition, five GB3 sequences isolated between 1996 and 1998 displayed the 60-nucleotide partial duplication of the G gene. These sequences did not group in a single cluster, but were present in four different clades. In addition, these GB3 strains showed nucleotide and amino acid differences compared with the initial BA strains described in Argentina in almost all genes. These observations suggest the occurrence of independent duplication events that, ultimately, resulted in the establishment of a dominant variant leading to the emergence of the BA genotype. This is consistent with previous reports that indicate that more than one duplication event resulted in new variants of human metapneumovirus and RSV-A^34–36^.

The definition of an early cluster of RSV-B strains as a distinct genotype (which we have termed GB0) is supported by the phylogenetic analysis, as well as the G gene intergenotypic p-distance analysis. The p-distance between the proposed early genotype and all other genotypes was higher than the value established as a threshold to identify a distinct genotype. In addition, analysis of all other RSV genes (except NS1 and N, for which there were two and one exceptions, respectively) supported the identification of these strains as a distinct genotype. Also, we identified 71 and 8 nucleotide and amino acid markers, respectively that are distinct for this genotype. These markers were found in 10 genes and 5 deduced proteins. Circulation of this genotype occurred in North America and Europe between 1972 and 1983.

Distinct molecular markers have been previously described for several RSV-B genotypes. For instance, BA13 had been reported to display unique amino acid changes (T232A, K233G, T240K/G, R242G, Q248R, D253G, T255A, T256A, K258G, D263Y and E292K)^12^. However, based on analysis of a large sequence dataset, we observed that many of these markers were not exclusive of BA13; for instance, R242G was also found to be present in GB6, and Q248R in GB4. Another example is BA9, which had been described as having two specific clusters named ATI and TRT based on substitutions at positions 107, 136, and 254 of the G protein (A107, T107, T136, R136, T254 and I254) and at positions 173 and 209 of the F protein (S173, L173, K209 and Q209)^37^; however, we observed that T107A and S172L are markers for BA-CC genotype and R136I for URU2. It is noteworthy that G protein amino acid substitutions T107A and T254I (markers of BA-CC), R136I (marker of URU2), and K258D/N (markers of GB4 and CB1-THB, respectively) may alter O-glycosylation patterns and, as a result, may affect antigenicity and facilitate homologous reinfections^37^. Nine unique amino acid markers were located at the F protein. However, none was found at the antigenic site targeted by palivizumab; in fact, all RSV-B F gene sequences included in the analysis were conserved at antigenic site II (aa 255–275). Five sequences (1.03% of the dataset), all corresponding to genotype BA that circulated between 2012–2014, showed the S276N substitution. Overall, we identified 1,213 nucleotide and 213 amino acid molecular markers. As previously noted, GB0 strains displayed eight unique amino acid molecular markers. In addition, this proposed early genotype had 185 nucleotide and 36 amino acid molecular markers which were shared with one or more genotypes.

Overall, our analysis allowed to identify molecular markers that at this time can be considered as specific of certain genotypes, particularly when several of them are identified together. While we identified certain markers with high specificity for a single genotype, many markers are shared by two or more genotypes. Therefore, it is likely that availability of more sequencing information, particularly from contemporaneous or future RSV strains, might modify the specificity estimations obtained by us. On the other hand, ongoing monitoring of the prevalence of these markers on currently circulating genotypes might help identity the emergence of new genotypes in the future.

Analysis of the temporal and geographical distribution of the different RSV-B genotypes showed that after the report of this RSV subgroup in 1960, two distinct genotypes circulated in North America and Europe, GB1 and the novel early genotype termed G0 in this report (Fig. 6). During the decade between 1980 and 1989 viral strains belonging to these genotypes continued to be the predominant RSV-B viruses, with the appearance of additional genotypes, namely JAB1-NZB2, GB2, SAB1, and URU2. Between 1990 and 1999 RSV-B strains displayed further diversification including GB3, GB4, and GB6; of note, viral strains with a partial duplication of the G gene emerged within the GB3 genotype (described as BA genotype by Trento et al.^33^) during this time. After the year 2000, further diversification of RSV-B strains led to the development of new genotypes with (BA, BA-C, and BA-CC genotypes) and without (CB1-THB) the partial duplication of the G gene. Interestingly, the temporal evolution of RSV-A strains has shown a similar pattern, although there have been fewer genotypes described, and the emergence of RSV-A strains with a partial duplication of the G gene (ON1 strains), analogous to the BA strains, occurred approximately ten years later^36,38^. This could be explained, in part, by the fact that RSV-B evolution rate is higher than that of RSV-A.

In summary, despite displaying a wide diversity, RSV-B strains can be grouped in 15 distinct genotypes. The 60-nt partial duplication of the G gene does not identify a unique genotype and includes viral strains within four different genotypes (GB3, BA, BA-C, and BA-CC). Finally, we have identified a previously unrecognized early genotype which we have termed as GB0, since it circulated prior to the emergence of the GB1 genotype.

## Materials and methods

### Dataset selection and curation

The dataset for this study included all RSV-B strains for which at least the complete G gene ectodomain sequence had been deposited in GenBank up until September 2018. All RSV sequences available on NCBI were downloaded and, as this study focused on the analysis of the complete ectodomain of circulating RSV strains, several inclusion criteria had to be met in order to proceed with subsequent analyses. Synthetic RSV sequences, sequences from organisms other than RSV, and sequences with nucleotide length smaller than the complete ectodomain length were excluded. 10,340 sequences fulfilled the inclusion criteria and were downloaded.

Blast2GO v5.2.5 software was used to analyze the sequences and identify the strains corresponding to RSV-B; local BLAST was carried out against a database of 10 RSV-B G gene ectodomain reference sequences resulting in 3,029 RSV-B G gene sequences^39^. These sequences were aligned using MAFFT v7.450 and, subsequently, manually inspected and aligned if needed using BioEdit v7.0.5.3^40,41^. The sequences were inspected and those that presented gaps (other than the partial 60 nt duplication described in 2000)^33^, degenerate nucleotides, insertions or deletions at the initial, middle or terminal ectodomain (with exception of a six nucleotide deletion present at position 473–478 in 27% of the sequences) were eliminated from the dataset to prevent genotype misassignment. 2,198 RSV-B complete G ectodomain sequences fulfilled the criteria previously described and, for each sequence, information such as accession number, strain, year and country of isolation was registered.

Two different alignments were obtained from this dataset: the first included only the second hypervariable region (spanning from nt 645 to the end of the G gene); the second alignment included the complete G gene ectodomain (spanning from nt 312 to the end of the G gene).

The complete dataset consisted of 2198 complete G gene ectodomain sequences, of which 18 sequences consisted of the complete G gene, 29 consisted of the complete G and SH genes, and 478 consisted of complete genome sequences; the 10 RSV genes were trimmed from 3′UTR to 5′UTR and aligned using MUSCLE algorithm and duplicated sequences were removed from each gene alignment to perform the cladistic analyses^42^.

### Reference sequences selection

Reference sequences were selected through an extensive search of the literature. First, we identified 736 sequences included in 22 articles published between 2003 to 2018 as genotype references^5,6,10,13–16,23–26,33,43–52^; subsequently, these were assessed to verify concordance in genotype assignment; some sequences could not be identified or traced due to assignment of IDs different from strain names or GenBank accession numbers. A total of 691 of these 736 sequences were traceable. Sequences that had been used by two or more of the 22 authors as reference sequences and those that have been identified as unique genotypes were selected, resulting in a total of 188 sequences (Supplementary Table 2). Sequences which had been assigned by two or more authors as representative of a different genotype were discarded (sequences with discordant genotype identity). In the case of recently or uniquely identified genotypes the first sequences submitted to NCBI were used as reference sequences. Genotype assignment agreed between two or more authors in 115 of 188 sequences; in addition, 64 sequences from recently or uniquely identified genotypes were included as references based only on their original description^5–7,10,13–16,18,19,23–26,33,44–50,52^. The length of 122 of the resulting 188 reference sequences was shorter than the complete G gene ectodomain. To resolve this limitation during genotype assignment of sequences included in the study dataset, we selected equivalent references using a Maximum Likelihood analysis of the G gene second hypervariable region under the GTR + Γ + I model and 1,000 bootstrap iterations, as previously described^8^. For all subsequent analyses, 169 original or equivalent reference sequences listed in Supplementary Table 3 were used.

### Genotype assignment

Topologies from Maximum Likelihood analysis under GTR + Γ + I model with 1,000 bootstrapping iterations inferred with MEGA X v10.0.3, as well as a Maximum Clade Credibility Tree generated from a Bayesian Skyline Plot Analysis inferred with BEAST v2.5.1 package were visualized on FigTree v1.4 and considered for genotype assignment by clade clustering with reference or equivalent sequences^53^. P-distance matrices were generated with MEGA X v10.0.3 to calculate intragenotypic and intergenotypic distance for the G gene ectodomain as well as the 10 complete genes sequences.

The Maximum Clade Credibility Tree was also used to recreate the history of major changes of RSV-B over time and was generated from unique complete G gene ectodomain sequences using TreeAnnotator v2.5.1 from the corresponding phylogenetic analysis by the MCMC method performed with BEAST v2.5.1 package. Bayesian Skyline method was used to analyze the dataset assuming both relaxed and strict molecular clock. MCMC were run 400,000,000 steps and sampled every 20,000 steps; convergence achievement was confirmed with Tracer v1.7.1.

All 10 RSV-B genes (NS1, NS2, N, P, M, SH, G, F, M2, and L) were characterized and analyzed via cladistic analysis and calculation of intragenotypic and intergenotypic p-distance matrices using MEGA X v10.0.3 software.

In addition, we carried out recombination analyses with the use of RDP4 v.4.100 to identify potential recombinants; the following algorithms were included in the analysis: RDP, GENECONV, BOOTSCAN, MaxChi, CHIMAERA, SISCAN, and 3SEQ^31^. In addition, the GARD algorithm was also used to confirm the presence of recombination events^32^.

### Molecular markers detection

To detect molecular markers distributed all along the 10 RSV-B genes, sequences were grouped according to the previously assigned genotype, sequences were aligned spanning from 3′UTR to 5′UTR and translated using BioEdit v7.0.5.3. Differences of each grouped genotype with respect to RSV-B reference sequence “strain B1” (accession number NC_001781.1) were recorded^54^; the differences were considered molecular markers if a nucleotide or amino acid shift occurred at a site with respect to the reference sequence in 75% or more of the genotyped sequences. This analysis included 478 complete genome sequences for 8 of 10 genes (NS1, NS2, N, P, M, F, M2, and L), 507 sequences for SH, and 525 for complete G gene.

## Supplementary Information

Supplementary Information.

## Data Availability

This study was carried out with data retrieved from GenBank. All data used is available in public databases.
