# Supplementary material for: Respiratory syncytial virus B sequence analysis reveals a novel early genotype
Source: Sci Rep. 2021 Feb 10;11:3452. doi: 10.1038/s41598-021-83079-2 (PMC7876121; doi:10.1038/s41598-021-83079-2)
Supplement: Supplementary file 1 — Supplementary Information. [file 41598_2021_83079_MOESM1_ESM.docx]

**Respiratory syncytial virus B sequence analysis reveals a novel early genotype**

Juan C. Muñoz-Escalante, Andreu Comas-García, Sofía Bernal-Silva, Daniel E. Noyola

**SUPPLEMENTARY FIGURES AND TABLES**

**Supplementary Figure 1.** Distribution of unique and duplicated RSV-B sequences that contain at least the complete G gene ectodomain. Some of the sequences had degenerate nucleotides or indels that could lead to genotype mis assignation and were eliminated. The remaining sequences were used to analyze each gene using unique or all sequences in order to generate genes cladistic analyses or to detect molecular markers. Specific sequence numbers for each analysis are described in the diagram.

**
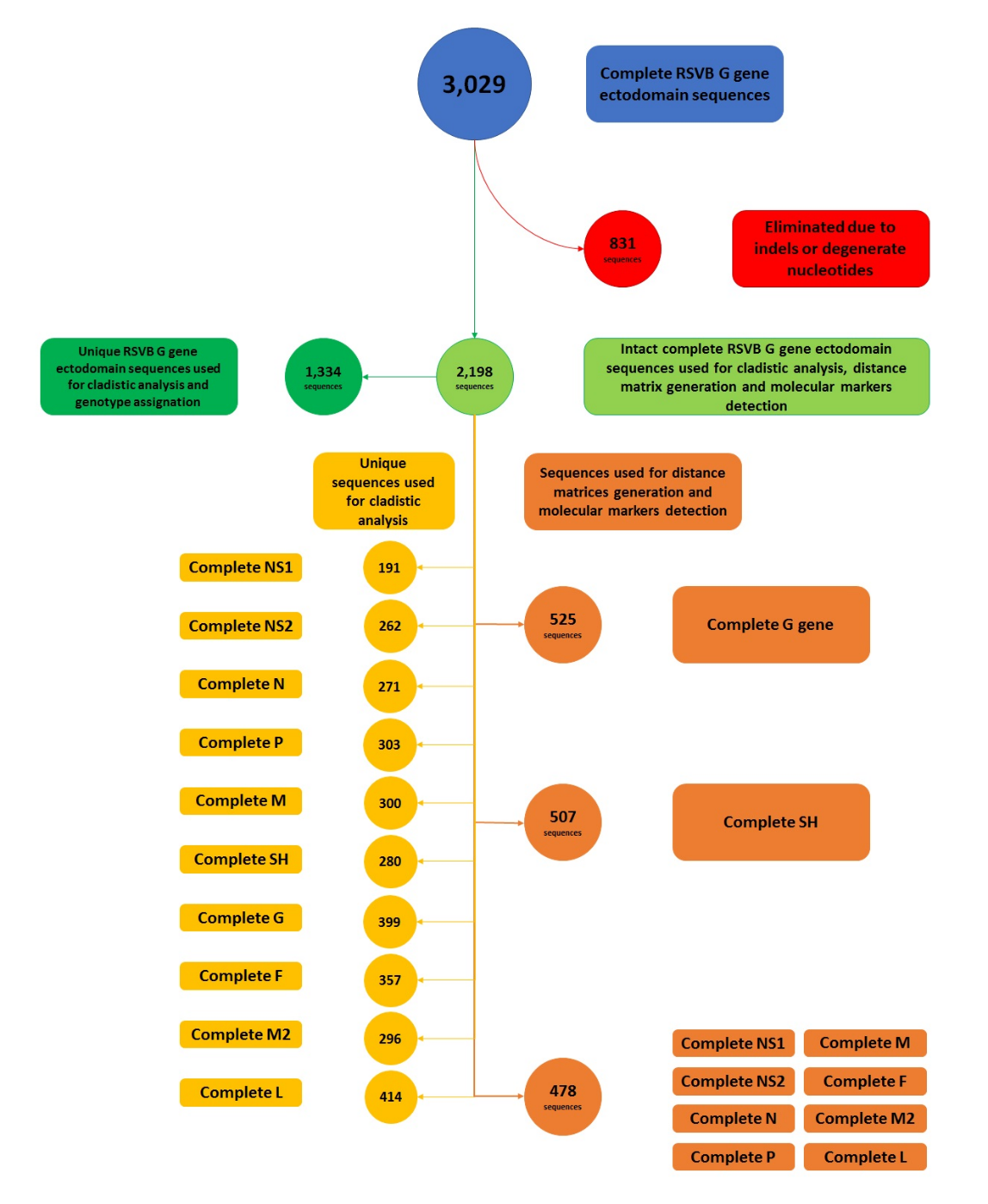
**

**Supplementary Figure 2.** Phylogenetic tree of 1,334 unique RSV-B complete G gene ectodomain sequences constructed by the Maximum Likelihood method. Genotype assignment was carried out with the use of 169 reference sequences including 37 previously described genotypes and prototype strains.

**
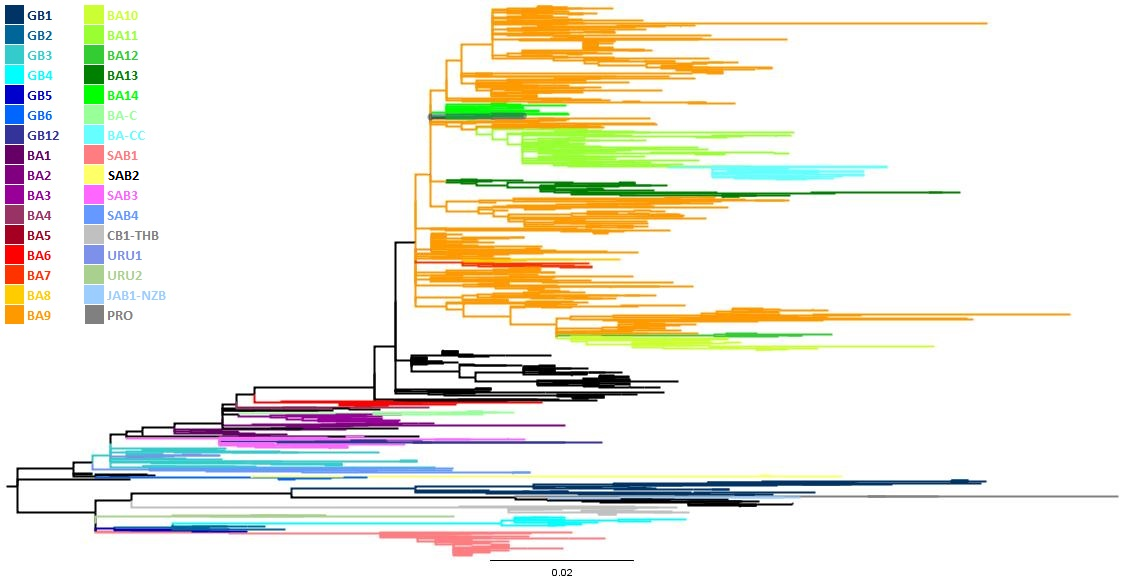
**

**Supplementary Figure 3.** Intergenotypic and intragenotypic p-distance analysis of 2,190 RSVB sequences assigned to previously described genotypes (represented in grey shade) and unidentified clades (represented in white). Intergenotypic distances lower than the threshold value (p=0.0358) are presented in red shade. Well defined subclades within genotypes or unidentified clades were analyzed as individual clades on initial analysis.


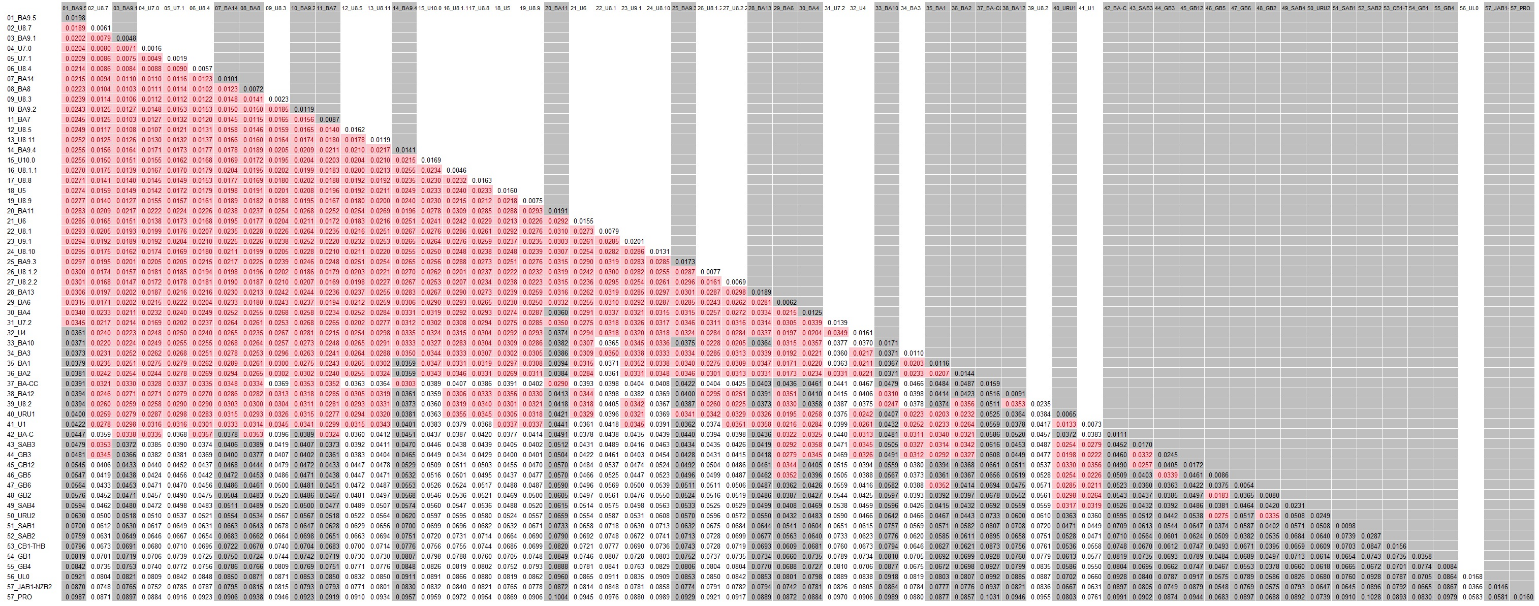


**Supplementary Figure 4.** Unrooted phylogenetic tree generated from 1,334 unique RSV-B complete G gene ectodomain sequences constructed by Maximum Likelihood analysis.


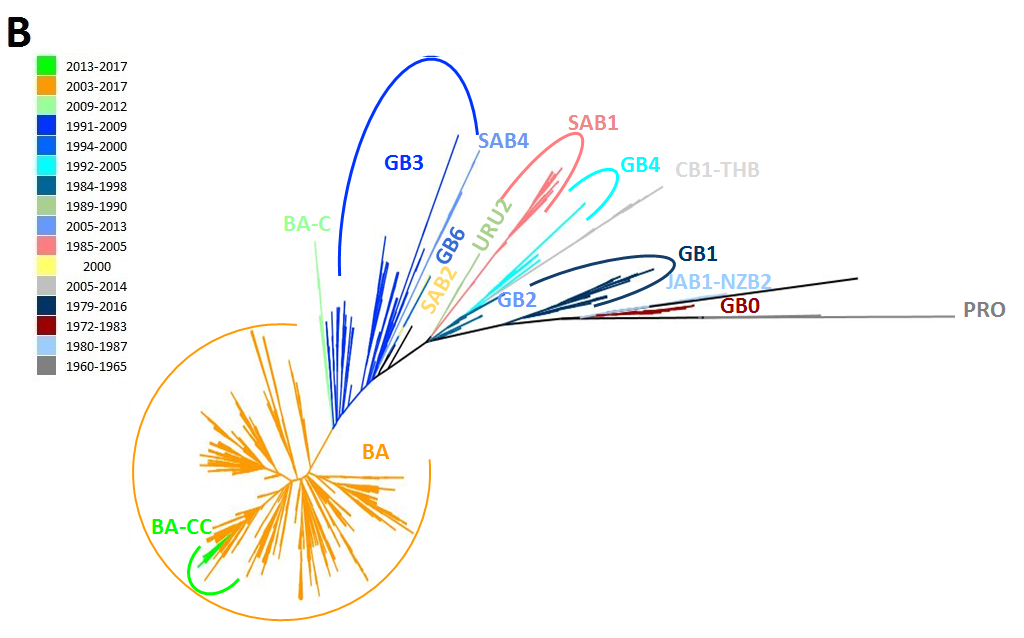


**Supplementary Table 1.** RSV-B genotype specific amino acid markers. The proportion of sequences for each genotype which present the specific amino acid marker are shown. In total 107 specific amino acid markers were identified in genotypes for which there are full genome sequences available. In addition, 11 markers were identified in genotypes for which only G gene (SAB2, CB1-THB) or G and SH genes (URU2) sequences are available.


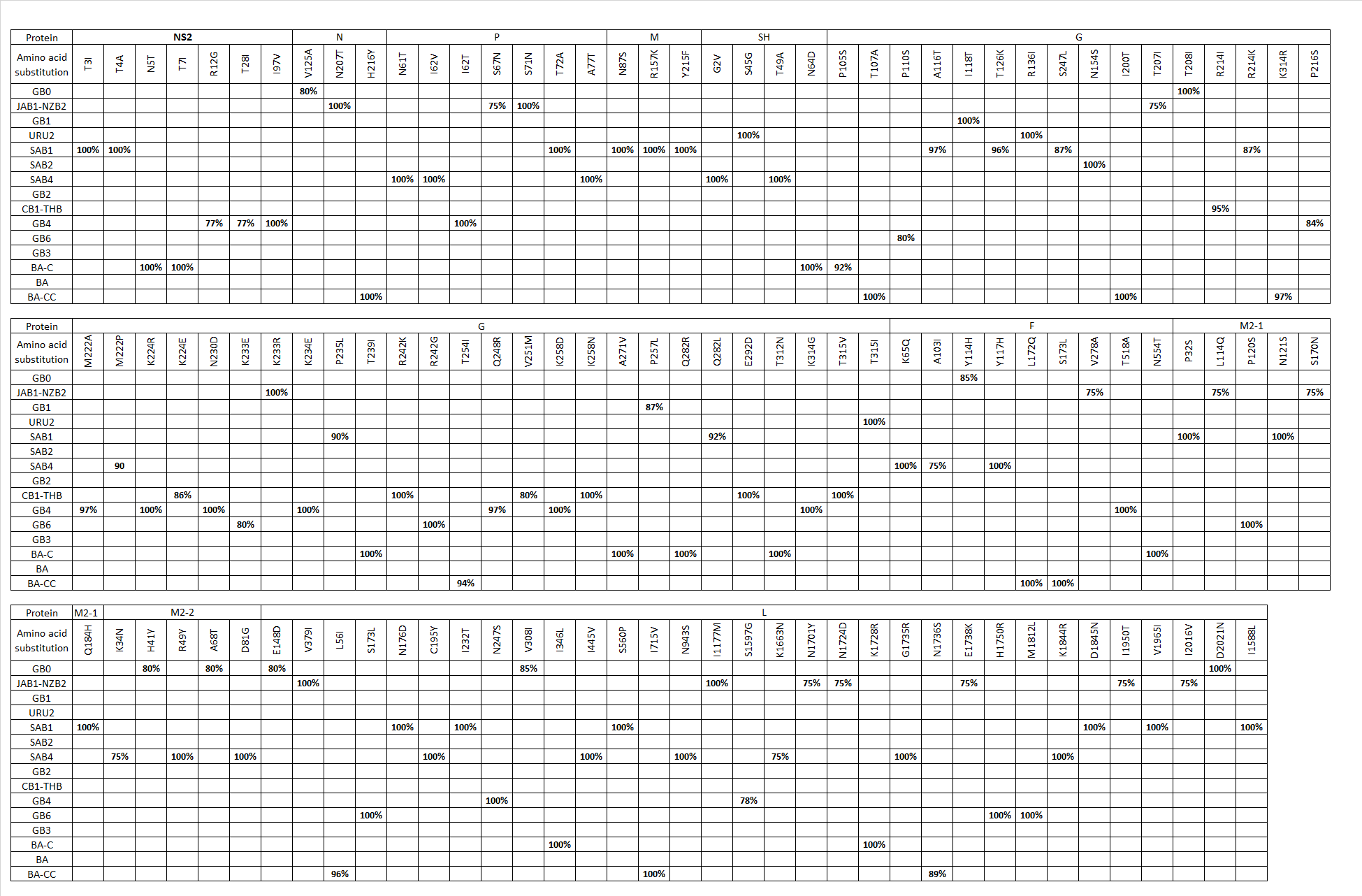


**Supplementary Table 2.** Revised RSV-B genotype reference sequences.

| Reference Sequences | |  | Reference Sequences | |  | Reference Sequences | |  | Reference Sequences | |
| --- | --- | --- | --- | --- | --- | --- | --- | --- | --- | --- |
|  |  |  |  |  |  |  |  |  |  |  |
| Accession | Genotype |  | Accession | Genotype |  | Accession | Genotype |  | Accession | Genotype |
| DQ227373.1 | BA1 |  | KX371866.1 | BA14 |  | HM459880.1 | BA9 |  | AB161389.1 | JAB1 |
| AY333364.1 | BA1 |  | KF300958.2 | BA14 |  | AB603469.1 | BA9 |  | AB161391.1 | JAB1 |
| DQ227364.1 | BA1 |  | KF300957.2 | BA14 |  | AB603467.1 | BA9 |  | AB161395.1 | JAB1 |
| DQ227363.1 | BA1 |  | KF300954.2 | BA14 |  | KF246629.1 | BA9 |  | AB161399.1 | JAB1 |
| DQ227374.1 | BA1 |  | DQ227389.1 | BA2 |  | KF246624.1 | BA9 |  | AB161392.1 | JAB1 |
| DQ227368.1 | BA1 |  | AY751119.1 | BA2 |  | HM459882.1 | BA9 |  | DQ171865.1 | NZB1 |
| HM459884.1 | BA10 |  | AY751122.1 | BA2 |  | HM459878.1 | BA9 |  | DQ171863.1 | NZB1 |
| HM459891.1 | BA10 |  | AY751121.1 | BA2 |  | AB603470.1 | BA9 |  | DQ171862.1 | NZB1 |
| HM459886.1 | BA10 |  | DQ227393.1 | BA2 |  | EU635867.1 | BA9 |  | DQ171864.1 | NZB1 |
| HM459890.1 | BA10 |  | AB175821.1 | BA2 |  | KF246607.1 | BA9 |  | DQ171845.1 | NZB2 |
| KC297426.1 | BA10 |  | AY751123.1 | BA2 |  | KC297486.1 | BA-C |  | DQ171842.1 | NZB2 |
| HM459888.1 | BA10 |  | DQ227377.1 | BA2 |  | KC297486.1 | BA-C |  | DQ171846.1 | NZB2 |
| HM459883.1 | BA10 |  | DQ227370.1 | BA3 |  | KC297456.1 | BA-C |  | DQ171841.1 | NZB2 |
| KP336524.1 | BA11 |  | DQ227375.1 | BA3 |  | KU254641.1 | BA-CCA |  | DQ171843.1 | NZB2 |
| KP336538.1 | BA11 |  | DQ227403.1 | BA3 |  | KU254642.1 | BA-CCA |  | DQ171844.1 | NZB2 |
| KP336543.1 | BA11 |  | DQ227397.1 | BA3 |  | KU254638.1 | BA-CCB |  | DQ171847.1 | NZB2 |
| KP336542.1 | BA11 |  | DQ227396.1 | BA4 |  | KU254643.1 | BA-CCB |  | M17213.1 | PRO |
| KP336527.1 | BA11 |  | DQ227407.1 | BA4 |  | KC297428.1 | CB1 |  | AF348825.1 | SAB1 |
| KP336537.1 | BA11 |  | HM459858.1 | BA4 |  | KC297471.1 | CB1 |  | JF704213.1 | SAB1 |
| KP336523.1 | BA11 |  | DQ227408.1 | BA4 |  | M73542.1 | GB1 |  | AY524573.1 | SAB1 |
| KP336526.1 | BA11 |  | HM459861.1 | BA4 |  | AF065250.1 | GB1 |  | AY660682.1 | SAB1 |
| KP336540.1 | BA11 |  | HM459863.1 | BA4 |  | M73540.1 | GB1 |  | AF348821.1 | SAB2 |
| KP336544.1 | BA11 |  | HM459860.1 | BA4 |  | AF013254.1 | GB1 |  | AF309678.1 | SAB2 |
| KP336533.1 | BA11 |  | AB175819.1 | BA5 |  | M73541.1 | GB1 |  | AF309676.1 | SAB2 |
| KP336541.1 | BA11 |  | AB175820.1 | BA5 |  | AY751256.1 | GB1 |  | AF348811.1 | SAB3 |
| KP336545.1 | BA11 |  | AB603483.1 | BA5 |  | AY751174.1 | GB12 |  | AF348813.1 | SAB3 |
| KP336528.1 | BA11 |  | AB603480.1 | BA5 |  | DQ171867.1 | GB13 |  | AF348812.1 | SAB3 |
| KP336532.1 | BA11 |  | AB603484.1 | BA5 |  | DQ171878.1 | GB13 |  | JN119987.1 | SAB4 |
| KP336534.1 | BA11 |  | AB603482.1 | BA5 |  | AF065251.1 | GB2 |  | JN120007.1 | SAB4 |
| KP336525.1 | BA11 |  | AY751117.1 | BA6 |  | DQ171858.1 | GB2 |  | JN119979.1 | SAB4 |
| KP336530.1 | BA11 |  | AY751111.1 | BA6 |  | DQ171849.1 | GB2 |  | JN119976.1 | SAB4 |
| KP336531.1 | BA11 |  | AY751105.1 | BA6 |  | AF233932.1 | GB3 |  | JN119989.1 | SAB4 |
| KP336535.1 | BA11 |  | AY751116.1 | BA6 |  | AF233929.1 | GB3 |  | KC342336.1 | THB |
| KP336536.1 | BA11 |  | HM459864.1 | BA7 |  | AF233933.1 | GB3 |  | KC342343.1 | THB |
| JX256977.1 | BA12 |  | HM459865.1 | BA7 |  | AF348817.1 | GB3 |  | AY488804.1 | URU1 |
| KF246585.1 | BA12 |  | AB603476.1 | BA7 |  | AF233931.1 | GB4 |  | AY488805.1 | URU1 |
| KF246586.1 | BA12 |  | AB470481.1 | BA7 |  | AY672698.1 | GB4 |  | AY333361.1 | URU2 |
| JX256976.1 | BA12 |  | HM459866.1 | BA7 |  | AY672691.1 | GB4 |  | AY488803.1 | URU2 |
| KX262621.1 | BA13 |  | HM459870.1 | BA7 |  | AF348824.1 | GB4 |  |  |  |
| KX262638.1 | BA13 |  | HM459868.1 | BA7 |  | AF233924.1 | GB4 |  |  |  |
| KX262625.1 | BA13 |  | HM459868.1 | BA7 |  | AF233928.1 | GB4 |  |  |  |
| KX262619.1 | BA13 |  | HM459868.1 | BA7 |  | AY751281.1 | GB5 |  |  |  |
| KF300960.2 | BA14 |  | AY751087.1 | BA7 |  | AY751280.1 | GB5 |  |  |  |
| KF300959.2 | BA14 |  | AB603477.1 | BA7 |  | AY751237.1 | GB6 |  |  |  |
| KF300955.2 | BA14 |  | HM459867.1 | BA7 |  | AY751239.1 | GB6 |  |  |  |
| KF300970.2 | BA14 |  | HM459871.1 | BA8 |  | AY751241.1 | GB6 |  |  |  |
| KF300952.2 | BA14 |  | AB470482.1 | BA8 |  | AB161387.1 | JAB1 |  |  |  |
| KX371867.1 | BA14 |  | HM459872.1 | BA8 |  | AB161390.1 | JAB1 |  |  |  |
| KF300953.2 | BA14 |  | HM459875.1 | BA8 |  | AB161388.1 | JAB1 |  |  |  |
| KX371868.1 | BA14 |  | HM459881.1 | BA9 |  | AB161386.1 | JAB1 |  |  |  |
|  |  |  |  |  |  |  |  |  |  |  |

**Supplementary Table 3.** RSV-B reference or equivalent reference sequences.

| Final Reference and Equivalent Sequences | | | |  | Final Reference and Equivalent Sequences | | | |
| --- | --- | --- | --- | --- | --- | --- | --- | --- |
| Accession | Genotype | Equivalent Accession | Author |  | Accession | Genotype | Equivalent Accession | Author |
| DQ227373.1 | BA1 | DQ227373.1 | 1 |  | HM459871.1 | BA8 | JX489454.1 | 27 |
| AY333364.1 | BA1 | AY333364.1 | 2 |  | AB470482.1 | BA8 | JX489456.1 | 27 |
| DQ227364.1 | BA1 | DQ227364.1 | 1 |  | HM459872.1 | BA8 | KC297452.1 | 6 |
| DQ227363.1 | BA1 | DQ227363.1 | 1 |  | HM459875.1 | BA8 | DQ227395.1 | 1 |
| DQ227374.1 | BA1 | DQ227374.1 | 1 |  | HM459881.1 | BA9 | JX645887.1 | 7 |
| DQ227368.1 | BA1 | DQ227368.1 | 1 |  | HM459880.1 | BA9 | DQ985143.1 | 21 |
| HM459884.1 | BA10 | KR816639.1 | 3 |  | AB603469.1 | BA9 | KF826822.1 | 28 |
| HM459891.1 | BA10 | KR816642.1 | 3 |  | AB603467.1 | BA9 | KC297484.1 | 6 |
| HM459886.1 | BA10 | KX655669.1 | 4 |  | KF246629.1 | BA9 | KF246629.1 | 17 |
| HM459890.1 | BA10 | AY751273.1 | 5 |  | KF246624.1 | BA9 | KF246624.1 | 17 |
| KC297426.1 | BA10 | KC297426.1 | 6 |  | HM459882.1 | BA9 | KP862424.1 | 25 |
| HM459888.1 | BA10 | JX645880.1 | 7 |  | HM459878.1 | BA9 | HQ731707.1 | 29 |
| HM459883.1 | BA10 | KC476943.1 | 8 |  | AB603470.1 | BA9 | JX645926.1 | 7 |
| KP336524.1 | BA11 | KC297473.1 | 6 |  | EU635867.1 | BA9 | KP862159.1 | 25 |
| KP336538.1 | BA11 | KU950682.1 | 9 |  | KF246607.1 | BA9 | KF246607.1 | 17 |
| KP336543.1 | BA11 | KJ939929.1 | 10 |  | KC297486.1 | BA-C | KC297486.1 | 6 |
| KP336542.1 | BA11 | KT781399.1 | 11 |  | KC297456.1 | BA-C | KC297456.1 | 6 |
| KP336527.1 | BA11 | KT781395.1 | 11 |  | KU254641.1 | BA-CCA | KX775822.1 | 12 |
| KP336537.1 | BA11 | KX775802.1 | 12 |  | KU254642.1 | BA-CCA | LC385004.1 | 14 |
| KP336523.1 | BA11 | KT781401.1 | 11 |  | KU254638.1 | BA-CCB | LC385008.1 | 14 |
| KP336526.1 | BA11 | KX765959.1 | 13 |  | KU254643.1 | BA-CCB | MF001058.1 | 30 |
| KP336540.1 | BA11 | KU950587.1 | 9 |  | KC297428.1 | CB1 | KC297428.1 | 6 |
| KP336544.1 | BA11 | KU950587.1 | 9 |  | M73542.1 | GB1 | M73542.1 | 31 |
| KP336533.1 | BA11 | KU950635.1 | 9 |  | AF065250.1 | GB1 | AF065250.1 | 32 |
| KP336541.1 | BA11 | KU950635.1 | 9 |  | M73540.1 | GB1 | M73540.1 | 31 |
| KP336545.1 | BA11 | LC385001.1 | 14 |  | AF013254.1 | GB1 | KY674983.1 | 30 |
| KP336528.1 | BA11 | LC385001.1 | 14 |  | M73541.1 | GB1 | M73541.1 | 31 |
| KP336532.1 | BA11 | KM586843.1 | 15 |  | AY751256.1 | GB1 | AY751256.1 | 5 |
| KP336534.1 | BA11 | KU950682.1 | 9 |  | AY751174.1 | GB12 | AY751174.1 | 5 |
| KP336525.1 | BA11 | KC297476.1 | 6 |  | DQ171867.1 | GB13 | KU316100.1 | 35 |
| KP336530.1 | BA11 | KX655681.1 | 4 |  | DQ171878.1 | GB13 | KP258742.1 | 33 |
| KP336531.1 | BA11 | KX655648.1 | 4 |  | AF065251.1 | GB2 | AF065251.1 | 32 |
| KP336535.1 | BA11 | MF443156.1 | 16 |  | DQ171858.1 | GB2 | KP258720.1 | 33 |
| KP336536.1 | BA11 | MF443156.1 | 16 |  | DQ171849.1 | GB2 | AY751250.1 | 5 |
| JX256977.1 | BA12 | KR816654.1 | 3 |  | AF233932.1 | GB3 | KP258742.1 | 33 |
| KF246585.1 | BA12 | KF246585.1 | 17 |  | AF233929.1 | GB3 | KP258702.1 | 33 |
| KF246586.1 | BA12 | KF246586.1 | 17 |  | AF348817.1 | GB3 | KP258745.1 | 33 |
| JX256976.1 | BA12 | KJ690605.1 | 18 |  | AF233931.1 | GB4 | JX198144.1 | 24 |
| KX262621.1 | BA13 | KF437511.1 | 19 |  | AY672698.1 | GB4 | AY751245.1 | 5 |
| KX262638.1 | BA13 | KX655686.1 | 4 |  | AY672691.1 | GB4 | AY751246.1 | 5 |
| KX262625.1 | BA13 | KX655686.1 | 4 |  | AF348824.1 | GB4 | KJ723484.2 | 34 |
| KX262619.1 | BA13 | KF437512.1 | 19 |  | AF233924.1 | GB4 | HQ731722.1 | 29 |
| KF300960.2 | BA14 | KF300960.2 | 20 |  | AF233928.1 | GB4 | KU316100.1 | 35 |
| KF300959.2 | BA14 | KF300959.2 | 20 |  | AY751281.1 | GB5 | AY751281.1 | 5 |
| KF300955.2 | BA14 | KF300955.2 | 20 |  | AY751280.1 | GB5 | AY751280.1 | 5 |
| KF300970.2 | BA14 | KF300970.2 | 20 |  | AY751237.1 | GB6 | AY751237.1 | 5 |
| KF300952.2 | BA14 | KF300952.2 | 20 |  | AY751239.1 | GB6 | AY751239.1 | 5 |
| KX371867.1 | BA14 | KX371867.1 | 20 |  | AY751241.1 | GB6 | AY751241.1 | 5 |
| KF300953.2 | BA14 | KF300953.2 | 20 |  | AB161387.1 | JAB1 | KU316115.1 | 35 |
| KX371868.1 | BA14 | KX371868.1 | 20 |  | AB161390.1 | JAB1 | KP258738.1 | 33 |
| KX371866.1 | BA14 | KX371866.1 | 20 |  | AB161388.1 | JAB1 | KP258738.1 | 33 |
| KF300958.2 | BA14 | KF300958.2 | 20 |  | AB161386.1 | JAB1 | KP258738.1 | 33 |
| KF300957.2 | BA14 | KF300957.2 | 20 |  | AB161389.1 | JAB1 | KU316136.1 | 35 |
| KF300954.2 | BA14 | KF300954.2 | 20 |  | AB161391.1 | JAB1 | KU316136.1 | 35 |
| DQ227389.1 | BA2 | DQ227389.1 | 1 |  | AB161395.1 | JAB1 | KU316136.1 | 35 |
| AY751119.1 | BA2 | AY751119.1 | 5 |  | AB161399.1 | JAB1 | KJ723485.2 | 34 |
| AY751122.1 | BA2 | AY751122.1 | 5 |  | AB161392.1 | JAB1 | KJ723485.2 | 34 |
| AY751121.1 | BA2 | AY751121.1 | 5 |  | DQ171845.1 | NZB2 | HQ731709.1 | 29 |
| DQ227393.1 | BA2 | DQ227393.1 | 1 |  | DQ171842.1 | NZB2 | HQ731709.1 | 29 |
| AB175821.1 | BA2 | AY751120.1 | 5 |  | DQ171846.1 | NZB2 | HQ731709.1 | 29 |
| AY751123.1 | BA2 | AY751123.1 | 5 |  | DQ171841.1 | NZB2 | HQ731709.1 | 29 |
| DQ227377.1 | BA2 | DQ227377.1 | 1 |  | DQ171843.1 | NZB2 | HQ731709.1 | 29 |
| DQ227370.1 | BA3 | DQ227370.1 | 1 |  | DQ171844.1 | NZB2 | HQ731709.1 | 29 |
| DQ227375.1 | BA3 | DQ227375.1 | 1 |  | DQ171847 | NZB2 | HQ731709.1 | 29 |
| DQ227403.1 | BA3 | DQ985154.1 | 21 |  | DQ171865.1 | NZB1 | KU316156.1 | 35 |
| DQ227397.1 | BA3 | DQ227397.1 | 1 |  | DQ171863.1 | NZB1 | KU316094.1 | 35 |
| DQ227396.1 | BA4 | DQ227396.1 | 1 |  | M17213.1 | PRO | JX198143.1 | 24 |
| HM459858.1 | BA4 | JX576761.1 | 22 |  | AF348825.1 | SAB1 | AY751272.1 | 5 |
| DQ227408.1 | BA4 | DQ270228.1 | 23 |  | AY524573.1 | SAB1 | KP317939.1 | 25 |
| AY751117.1 | BA6 | AY751117.1 | 5 |  | AY660682.1 | SAB1 | AY660682.1 | 36 |
| AY751111.1 | BA6 | AY751111.1 | 5 |  | AF348821.1 | SAB2 | AY327815.1 | 37 |
| AY751105.1 | BA6 | AY751105.1 | 5 |  | AF309678.1 | SAB2 | AY327815.1 | 37 |
| AY751116.1 | BA6 | AY751116.1 | 5 |  | AF309676.1 | SAB2 | AY327815.1 | 37 |
| HM459864.1 | BA7 | JX198152.1 | 24 |  | AF348811.1 | SAB3 | AY751163.1 | 5 |
| HM459865.1 | BA7 | KP862155.1 | 25 |  | AF348813.1 | SAB3 | AY751150.1 | 5 |
| AB603476.1 | BA7 | KP862390.1 | 25 |  | AF348812.1 | SAB3 | KP258724.1 | 33 |
| AB470481.1 | BA7 | AY751093.1 | 5 |  | JN119987.1 | SAB4 | KC297478.1 | 6 |
| HM459866.1 | BA7 | KC297492.1 | 6 |  | JN120007.1 | SAB4 | KC297430.1 | 6 |
| HM459870.1 | BA7 | JX908845.1 | 26 |  | JN119979.1 | SAB4 | DQ270231.1 | 23 |
| HM459868.1 | BA7 | KP862175.1 | 25 |  | JN119989.1 | SAB4 | KR816642.1 | 3 |
| HM459868.1 | BA7 | KC297492.1 | 6 |  | AY488804.1 | URU1 | AY751230.1 | 5 |
| HM459868.1 | BA7 | KC297492.1 | 6 |  | AY488805.1 | URU1 | AY751228.1 | 5 |
| AY751087.1 | BA7 | AY751087.1 | 5 |  | KC297471.1 | THB | KC297471.1 | 6 |
| AB603477.1 | BA7 | AY751087.1 | 5 |  | AY333361.1 | URU2 | AY333361.1 | 2 |
| HM459867.1 | BA7 | DQ985136.1 | 21 |  | | | | |

**1.** Trento A, Viegas M, Galiano M, et al. Natural history of human respiratory syncytial virus inferred from phylogenetic analysis of the attachment (G) glycoprotein with a 60-nucleotide duplication. *J Virol*. 2006;80(2):975-984.**2.** Trento A, Galiano M, Videla C, et al. Major changes in the G protein of human respiratory syncytial virus isolates introduced by a duplication of 60 nucleotides. *J Gen Virol*. 2003;84(Pt 11):3115-3120. **3.** Nilwong,O., Bhattarakosol,P. and Kowitdamrong,E. Direct submission **4.** Shabman,R., Das,S.R., Puri,V., Fedorova,N., Amedeo,P., Williams,M., Shrivastava,S. and Halasa,N. Direct submission **5.** Zlateva KT, Lemey P, Moës E, Vandamme AM, Van Ranst M. Genetic variability and molecular evolution of the human respiratory syncytial virus subgroup B attachment G protein. *J Virol*. 2005;79(14):9157-9167. **6.** Cui G, Zhu R, Qian Y, et al. Genetic variation in attachment glycoprotein genes of human respiratory syncytial virus subgroups a and B in children in recent five consecutive years. *PLoS One*. 2013;8(9):e75020. **7.** Houspie L, Lemey P, Keyaerts E, et al. Circulation of HRSV in Belgium: from multiple genotype circulation to prolonged circulation of predominant genotypes. *PLoS One*. 2013;8(4):e60416. **8.** Pretorius MA, van Niekerk S, Tempia S, et al. Replacement and positive evolution of subtype A and B respiratory syncytial virus G-protein genotypes from 1997-2012 in South Africa. *J Infect Dis*. 2013;208 Suppl 3:S227-S237. **9.** Das,S.R., Halpin,R.A., Shilts,M., Puri,V., Akopov,A., Fedorova,N., Stockwell,T., Amedeo,P., Bishop,B., Katzel,D., Schobel,S., Shrivastava,S. and Hartert,T. Direct submission **10.** Do LAH, Wilm A, van Doorn HR, et al. Direct whole-genome deep-sequencing of human respiratory syncytial virus A and B from Vietnamese children identifies distinct patterns of inter- and intra-host evolution. *J Gen Virol*. 2015;96(12):3470-3483. **11.** Fan,R.Y., Qu,X.W. and Fan,C.P. Direct submission **12.** Kamau,E.M., Agoti,C.N., Lewa,C.S., Cane,P.A., Bett,A., Medley,G.F. and Nokes,J.D. Direct submission **13.** Shabman,R., Das,S.R., Shilts,M., Fedorova,N., Puri,V., Shrivastava,S., Amedeo,P., Williams,M., Barratt,K., Mitchell,J. and Jennings,L. Direct submission **14.** Okamoto M, Sakamoto M, Dapat C, et al. Complete Genome Sequences of 12 Human Respiratory Syncytial Virus (*Human Orthopneumovirus*) Strains Detected in Children with Repeated Subgroup B Infections in the Philippines. *Microbiol Resour Announc*. 2018;7(22):e01017-18. **15.** Xie,J.H., Zhu,B., Zhong,J.Y., Chen,Y. and Zhang,Y.Y. Direct submission **16.** Trento,A., Rodriguez-Fernandez,R., Gonzalez,M.I., Gonzalez,F., Mas,V., Vazquez,M., Palomo,C. and Melero,J.A. Direct submission **17.** Choudhary ML, Anand SP, Wadhwa BS, Chadha MS. Genetic variability of human respiratory syncytial virus in Pune, Western India. *Infect Genet Evol*. 2013;20:369-377. **18.** Raghuram,V.S., Khan,W.H., Broor,S. and Parveen,S. Direct submission **19.** Bashir U, Alam MM, Sadia H, Zaidi SS, Kazi BM. Molecular characterization of circulating respiratory syncytial virus (RSV) genotypes in Gilgit Baltistan Province of Pakistan during 2011-2012 winter season PLoS One. 2016;11(3):e0151009. **20.** Abrego,L., Delfraro,A., Franco,D., Castillo,C., Cano,M., Castillo,M., Castillo,J., Pascale,J. and Arbiza,J. Direct submission **21.** Zlateva KT, Vijgen L, Dekeersmaeker N, Naranjo C, Van Ranst M. Subgroup prevalence and genotype circulation patterns of human respiratory syncytial virus in Belgium during ten successive epidemic seasons. *J Clin Microbiol*. 2007;45(9):3022-3030. **22.** Tan L, Coenjaerts FE, Houspie L, et al. The comparative genomics of human respiratory syncytial virus subgroups A and B: genetic variability and molecular evolutionary dynamics. *J Virol*. 2013;87(14):8213-8226. **23.** Deng,J., Zhu,R., Qian,Y., Zhao,L. and Wang,F. Direct submission **24.** Tapia,L.I. and Piedra,P.A. Direct submission **25.** Agoti CN, Otieno JR, Munywoki PK, et al. Local evolutionary patterns of human respiratory syncytial virus derived from whole-genome sequencing. *J Virol*. 2015;89(7):3444-3454. **26.** Machado,D.B.B., Motta,F.C., Souza,T.M.L., Mesquita,M.M. and Siqueira,M.M. **27.** Moura,F.E.A., Thomazelli,L.M., Candido,E.D.O., Florencio,C.M.G.D., Pereira,S.A.R., Oliveira,F.M.S., Alves,A.A., Ocadaque,C.J. and Durigon,E.L. Direct submission **28.** Lorenzi,H., Town,C., Halpin,R., Bera,J., Ransier,A., Fedorova,N., Stockwell,T., Amedeo,P., Appalla,L., Bishop,B., Edworthy,P., Gupta,N., Hoover,J., Katzel,D., Li,K., Schobel,S., Shrivastava,S., Thovarai,V., Wang,S., Rebuffo-Scheer,C., Fan,J., He,J., Kehl,S.C., Lederboer,N., Jurgens,L.A., Bose,M.E., Beck,E.T., Kumar,S., Wentworth,D.E. and Henrickson,K.J. Direct submission **29.** Gaunt ER, Jansen RR, Poovorawan Y, Templeton KE, Toms GL, Simmonds P. Molecular epidemiology and evolution of human respiratory syncytial virus and human metapneumovirus. *PLoS One*. 2011;6(3):e17427. **30.** Greninger,A.L., Makhsous,N., Kuypers,J., Shean,R.C. and Jerome,K.R. Direct submission **31.** Sullender WM, Mufson MA, Anderson LJ, Wertz GW. Genetic diversity of the attachment protein of subgroup B respiratory syncytial viruses. *J Virol*. 1991;65(10):5425-5434. **32.** Peret TC, Hall CB, Schnabel KC, Golub JA, Anderson LJ. Circulation patterns of genetically distinct group A and B strains of human respiratory syncytial virus in a community. *J Gen Virol*. 1998;79 ( Pt 9):2221-2229. **33.** Das,S.R., Halpin,R.A., Puri,V., Akopov,A., Fedorova,N., Stockwell,T., Amedeo,P., Bishop,B., Katzel,D., Schobel,S., Shrivastava,S., Wentworth,D.E. and Caserta,M. Direct submission **34.** Das,S.R., Halpin,R.A., Puri,V., Akopov,A., Fedorova,N., Tsitrin,T., Stockwell,T., Amedeo,P., Bishop,B., Gupta,N., Hoover,J., Katzel,D., Schobel,S., Shrivastava,S., Wentworth,D.E. and Caserta,M. Direct submission **35.** Das,S.R., Halpin,R.A., Puri,V., Akopov,A., Fedorova,N., Stockwell,T., Amedeo,P., Bishop,B., Katzel,D., Schobel,S., Shrivastava,S., Hall,C.B., Tesini,B.L., Schnabel,K.C., Walsh,E.E. and Caserta,M. Direct submission **36.** Scott PD, Ochola R, Ngama M, et al. Molecular epidemiology of respiratory syncytial virus in Kilifi district, Kenya. *J Med Virol*. 2004;74(2):344-354. **37.** Althani,A.A. and Zambon,M. Direct Submission
